# A mathematical framework for analysing particle flow in a network with multiple pools

**DOI:** 10.1098/rsos.231588

**Published:** 2024-05-08

**Authors:** Aditi Jain, Arvind Kumar Gupta

**Affiliations:** ^1^ Department of Mathematics, Indian Institute of Technology Ropar, Rupnagar, Punjab 140001, India

**Keywords:** network with multiple pools, ribosome flow model, cooperative dynamical system, first integral, entrainment

## Abstract

In many real-world systems, the entry rate of particles into a lane is affected by the occupancy of nearby pools. For instance, in biological networks, the concentration of molecules on the side of a membrane affects the entry of particles through the membrane. To understand the behaviour of such networks, we develop a network model of ribosome flow models (RFMs) having multiple pools where each RFM captures the dynamics of particle flow in a lane and competes for the finite resources present at the nearby pool. We study a ribosome flow model network with two pools (RFMNTP) and show that the network always admits a steady state. We then analyse the behaviour of the RFMNTP with respect to modifying the transition rate through a theoretical framework. Simulations of the RFMNTP demonstrate a counterintuitive result. For example, increasing any of the transition rates in the presence of a slow site in an RFM can increase the output rate of some RFMs and decrease the output rate of the other RFMs simultaneously. This suggests that the role of local sharing of particles incorporated is non-trivial. Finally, we illustrate how these results can provide insights into studying a network with multiple pools.

## 1. Introduction

Movement is a vital aspect of life. Various cellular and physical processes involve the movement of particles along tracks. These processes generally take place in parallel, and they compete for the available limited resources. For example, during gene expression, all DNA (mRNA) molecules simultaneously compete for the limited amount of RNAPs (ribosomes) [1–3]. This competition generates a network in which an indirect coupling induces interactions among the lanes even in the absence of explicit connections [4,5]. Hence, it is of considerable interest to analyse such networks in the presence of these interactions and also to design several resource-sharing synthetic gene networks [6,7]. In physical systems such as vehicular flow, the number of vehicles moving along the roads is finite. The entry rate of the vehicles along the road is affected owing to the queue of vehicles waiting to enter a road, where each vehicle competes with other vehicles for limited space on the road [8]. This requires modelling the complex road network to comprehend the traffic flow, thereby reducing travel time and preventing traffic deadlocks [9–11].

Several computational and mathematical models have been developed to study resource-sharing networks [12–14]. One such model includes a set of totally asymmetric simple exclusion processes (TASEPs) that are interconnected to each other via a pool of free particles [15–17]. In TASEP, a chain of sites models the one-dimensional track and particles hop unidirectionally, with some probability, from one site to the next site only if the target site is empty [18,19]. The model encapsulates the simple exclusion concept through the fact that particles have volume and are unable to pass a moving particle ahead. The TASEP and its networks have been used to model and analyse various natural and artificial systems, including mRNA translation, vehicular traffic flow and more [10,20,21]. Regardless of its simple description, rigorous analysis of networks of TASEPs is complex, exact solutions exist for simplified cases and most non-homogeneous cases are studied via numerical methods or extensive Monte Carlo simulations [22–24]. Hence, understanding the effect of parameters on the dynamics of a system through TASEP-based models has proved challenging. To allow analytical treatment, network models based on ribosome flow models (RFMs) were developed [25–27].

The RFM is a deterministic model that is derived via the mean-field approximation of TASEP [28,29]. It is a nonlinear model, and yet, it is very amenable to mathematical analysis using methods from systems and control theory, in particular, contraction theory [30–32], monotone dynamical systems [33,34], theory of continued fractions [35], etc. The TASEP-based models use mean-field approximations in the thermodynamic limit, and hence, analysis of the TASEP becomes accurate only when the number of sites n goes to infinity, while the analysis of the RFM provides results that are valid for every n . The theory of TASEP focuses on domain wall theory, phase transitions, etc. [36–38], which is different from the theoretical approach used to analyse RFM. The RFM and its generalizations have been developed to understand the complex dynamics of a variety of transport phenomena on a single lane, including translation [35,39–44], transcription [45], motor protein traffic [46,47], phosphorelay [48] and more. There are lattice hydrodynamic models that use ordinary differential equations to model the flow of vehicles along the lanes [49–51]. Therefore, the framework of RFM that also describes the flow of particles can serve as the basis for understanding the dynamics of vehicular traffic.

A network model called the ribosome flow model network with a finite pool (RFMNP) includes several RFMs that are interconnected to each other via a single pool of free particles [25]. It analyses the behaviour of such networks where the entry rate of the particles on lanes is affected by a single pool, that is, a single pool feeds all the input to the RFMs, and the output of each RFM is fed again into the pool. The number of particles remains conserved in this network and the network properties have been rigorously studied using tools from cooperative dynamical systems with a first integral [52–54]. An RFM network in [26] is a generalized network that analyses various network topologies using a set of interconnected RFMs. It models the static connections between the RFMs, and hence, the input to each RFM is a source (maybe pools of free ribosomes in the cell) whose output rate is a constant or proportion of the output of other RFMs. Here, the pool supplies a constant input source, and hence, it does not take into account the competition effects on the network’s behaviour owing to finite resources.

Almost all prior research has provided an understanding of biological activities constrained by a single pool. However, taking into account the concept of multiple pools helps one to comprehend the participation of particles in the vicinity of their targets. For instance, in the context of introducing synthetic circuit genes, a network model called orthogonal ribosome flow model (ORFM) was introduced, where the ribosome pool has been divided by the use of orthogonal ribosomes, and the introduced genes are only translated by mutated ribosomes [55]. The concept of orthogonal ribosomes was used to increase the protein output by decoupling circuit genes from the host pool of ribosomes. The concept of multiple pools also provides a useful framework to model vehicular flow between different cities where each pool represents each city.

In this article, we study the idea that the entry rate of the particles on lanes is affected by the occupancy of the nearby pool. Studying a minimal model of a two-pool network is a useful strategy for gaining insights into the behaviour of more complex systems with multiple pools. We present our theoretical investigation of a two-pool network and then illustrate how one can generalize it to study a network with multiple pools. We introduce a new model called the ribosome flow model network with two pools (RFMNTP) that include several RFMs interconnected via two dynamical pools of free particles. It captures the feature that the particles located far away from RFMs will not impact the initiation rates of these RFMs. Therefore, each RFM can be associated with two pools of particles, one pool containing particles impacting the initiation rate of the RFM and the other pool receiving its output. The whole system being closed conserves the number of particles. This two-pool network then models vehicular flow between the two cities where each pool represents each city. Similarly, pedestrian flow involving the movement of people between two places is reminiscent of the fact that it can be studied by incorporating two pools in the network.

By using the theory of cooperative dynamical systems with a first integral, we prove that the RFMNTP admits a continuum of steady-state points [52–54]. Therefore, the same steady-state point is attained by any two solutions starting from initial conditions corresponding to an equal total number of particles in the network, and hence, the network can be analysed by the steady-state density profile. This theoretical analysis can also be easily extended to prove the stability results for multiple pools. Next, we study how a change in one RFM affects the dynamics of the network. The change in the steady state with respect to changing the rate of a site in a specific RFM, say R , can be any one of the following outcomes: (i) steady-state pool densities can both increase (decrease) simultaneously, (ii) a decrease in the steady-state pool density that is feeding R and an increase in the steady-state density of the other pool. The results hold for any set of parameters in the network.

The structure of this article is organized as follows. The next section reviews the RFM and introduces the network of RFMs with multiple pools. A two-pool network and our main mathematical results are described in §3. The idea of understanding the dynamics of a network with multiple pools is illustrated in §4. The final section summarizes and suggests some directions for further research. The proofs of the results are provided in the Appendix for ease of reading. The source code which simulates differential equations described in this article can be accessed on GitHub at the following link: https://github.com/aditi081/matlab-codes-Networks-of-RFMs-with-multiple-pools.git.

## 2. The mathematical framework

Since the building blocks of our network model are RFMs, we first review RFM briefly.

### 2.1. The ribosome flow model

**Figure 1.**
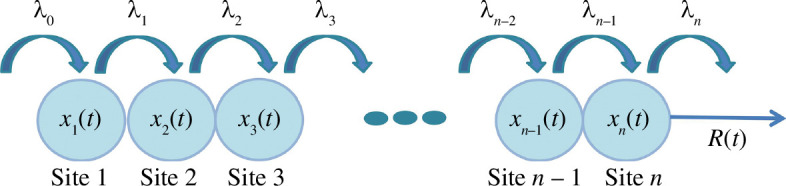
The RFM models unidirectional flow along a lattice of n sites. The parameter $\lambda_{i}>0$ represents the transition rate from site i to site i+1 , where $\lambda_{0}$ ( $\lambda_{n}$ ) represents the initiation (exit) rate. The state variable $x_{i}(t)$ represents the density of site i at time t . The output rate at any time t is R(t) .

The RFM is a continuous-time model for the unidirectional flow of particles along a lattice of n consecutive sites. The state variable $x_{j}(t)$ , where j∈{1,2,…,n} represents the normalized particle density at site j at time t , where $x_{j}(t)=0\;[x_{j}(t)=1]$ represents that site j is totally empty [full]. The RFM is characterized by n+1 positive rates $\lambda_{j}$ , where j∈{0,1,…,n} . In particular, the entry [exit] rate of particles is controlled by $\lambda_{0}$ [ $\lambda_{n}$ ]. The rate of flow from site j to site j+1 is given by $\lambda_{j}x_{j}(1-x_{j+1})$ , that is, the ribosomes are more likely to flow from site j to site j+1 when $x_{j}$ is full and $x_{j+1}$ is empty. The output rate of exit of particles is $R(t):=\lambda_{n}x_{n}(t)$ . Figure 1 shows the topology of the RFM. The RFM is given by the following system of n first-order ordinary differential equations (ODEs):


(2.1) $$\begin{array}{ll} {\dot{x}}_{1} & =\lambda_{0}(1-x_{1})-\lambda_{1}x_{1}(1-x_{2}),\\ {\dot{x}}_{2} & =\lambda_{1}x_{1}(1-x_{2})-\lambda_{2}x_{2}(1-x_{3}),\\ & \vdots \\ {\dot{x}}_{n} & =\lambda_{n-1}x_{n-1}(1-x_{n})-\lambda_{n}x_{n}. \end{array}$$

The trajectories of the RFM evolve on the n -dimensional unit cube denoted $C:=[0,1{]}^{n}$ , and it admits a unique steady-state density e in Int( C ), where Int( C ) denotes the interior of C [29]. This mathematical analysis is based on the theory of cooperative dynamical systems [33,34]. In [40], it has been shown that there exists a spectral representation for the mapping from $\lambda_{0}$ , $\lambda_{1}$ , … , $\lambda_{n}$ to steady-state e given by (n+2)×(n+2) Jacobi matrix


$$A:=\left[\begin{array}{ccccc} 0 & \lambda_{0}^{-1/2} & 0 & \ldots & 0\\ \lambda_{0}^{-1/2} & 0 & \lambda_{1}^{-1/2} & \ldots & 0\\ 0 & \lambda_{1}^{-1/2} & 0 & \ldots & 0\\ & & \vdots \\ 0 & \ldots & 0 & 0 & \lambda_{n}^{-1/2}\\ 0 & \ldots & 0 & \lambda_{n}^{-1/2} & 0 \end{array}\right].$$

Note that A is a non-negative, symmetric and irreducible matrix, and hence, it admits a unique maximal eigenvalue σ>0 , and the entries of a corresponding eigenvector $\zeta \in R^{n+2}$ are all positive for all i∈{1,2,…,n+2} [56]. It has also been proved in [40] that the steady-state values of the RFM satisfy


(2.2) $$e_{j}=\frac{\zeta_{j+2}}{\lambda_{j}^{1/2}\sigma \zeta_{j+1}},\;\;\text{for}\;\;j=1,2,\ldots ,n,$$

and the steady-state output rate satisfies


(2.3) $$R=\frac{1}{\sigma^{2}}.$$

To build a network of interconnected RFMs, the first step is to extend the RFM into a single-input–single-output (SISO) system. This is done by adding a time-varying measurable and bounded function $v:R_{+}⟶R_{+}$ to the RFM [39], where $R_{+}=R^{+}\cup \{0\}$ . In the context of translation, the function v represents the flow of ribosomes into the mRNA from the cell environment. The equations describing the RFM with the input and the output are as follows:


(2.4) $$\begin{array}{ll} {\dot{x}}_{1} & =\lambda_{0}v(1-x_{1})-\lambda_{1}x_{1}(1-x_{2}),\\ {\dot{x}}_{2} & =\lambda_{1}x_{1}(1-x_{2})-\lambda_{2}x_{2}(1-x_{3}),\\ & \vdots \\ {\dot{x}}_{n} & =\lambda_{n-1}x_{n-1}(1-x_{n})-\lambda_{n}x_{n}, \end{array}$$

and $R(t)=\lambda_{n}x_{n}(t)$ .

The system given by equation (2.4) is a monotone control system [57]. It can also be seen that C is an invariant set of the dynamics, that is, any trajectory emanating from any point in C shall remain in it for all time t≥0 .

### 2.2. Network of ribosome flow models with multiple pools

**Figure 2.**
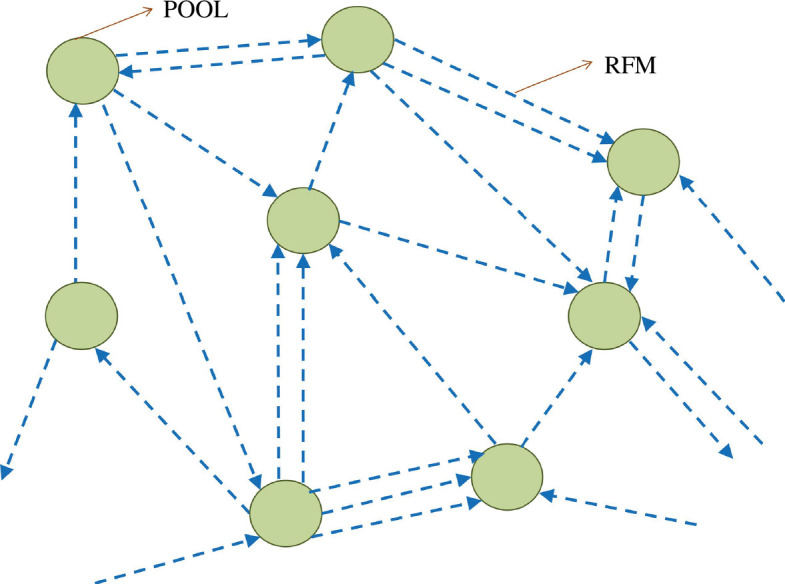
The graph representation of the network with multiple pools where each node (circle) represents the pool and the directed edges (dashed lines having arrows) represent a chain of sites on which particles undergo RFM dynamics. The arrow of the edge pointing to the pool represents that the output of the RFM is feeding the pool.

Now, we provide some insights into the dynamics of the flow of particles on several lanes connected via multiple pools. Consider a network consisting of M pools and N RFMs. Each pool is connected to at least one RFM that receives its input from the pool and at least one other RFM that feeds its output to the pool. The particles that are not attached to any RFM are present in the pools. The topology of this network can be represented using a directed multigraph where each node represents the pool and the directed edges represent the RFMs that indicate the flow of particles from one pool to the other pool (see figure 2).

Let RFM #i be described by the tuple $A_{i}:=\{n_{i},G_{i},x_{j}^{i},\lambda_{k}^{i}\}$ for i=1,2,…,N , $j=1,2\ldots ,n_{i}$ , $k=0,1,\ldots ,n_{i}$ , where $n_{i}$ is the dimension of RFM $\#i;G_{i}:R_{+}⟶R_{+}$ is the input function of RFM $\#i;x_{j}^{i}$ s are the state variables and $\lambda_{k}^{i}$ s are the positive transition rates along RFM #i . Let Pool #j density be described by $z_{j}(t)$ for j=1,2,…,M , where $z_{j}$ represents the average number of particles in the pool. Assume that Pool #j is feeding the input to RFM #k , k∈I , where I is a subset of the set {1,2,…,N} and let RFM $\#k^{\prime }$ , $k^{\prime }\in I^{\prime }$ , where $I^{\prime }$ is a subset of the set {1,2,…,N}∖I , is feeding its output to the Pool #j . Thus, the dynamics of RFM #k is described by the following ODEs:


(2.5) $$\begin{array}{rl} {\dot{x}}_{1}^{k} & =\lambda_{0}^{k}G_{k}(z_{j})(1-x_{1}^{k})-\lambda_{1}^{k}x_{1}^{k}(1-x_{2}^{k}),\\ {\dot{x}}_{2}^{k} & =\lambda_{1}^{k}x_{1}^{k}(1-x_{2}^{k})-\lambda_{2}^{k}x_{2}^{k}(1-x_{3}^{k}),\\ & \vdots \\ {\dot{x}}_{n_{k}}^{k} & =\lambda_{n_{k}-1}^{k}x_{n_{k}-1}^{k}(1-x_{n_{k}}^{k})-\lambda_{n_{k}}^{k}x_{n_{k}}^{k}, \end{array}$$

and the dynamics is described by the following balance equation for each Pool #j :


(2.6) $${\dot{z}}_{j}=\sum_{k^{\prime }\in I^{\prime }}\lambda_{n_{k^{\prime }}}^{k^{\prime }}x_{n_{k^{\prime }}}^{k^{\prime }}-\sum_{k\in I}\lambda_{0}^{k}G_{k}(z_{j})(1-x_{1}^{k}).$$

The entry rate of particles into the RFMs is modulated by the occupancy level of the nearby pool. Note that there is no direct link between the RFMs in the network, and the interconnections are via the pool of particles. The pool outflow functions $G_{k}$ describe the likelihood that the particles will attach to the RFMs. In other words, these functions model the competition for particles between the RFMs. Therefore, RFMs having a more effective initiation rate $\lambda_{0}^{k}G_{k}(z_{j})$ have more influx of particles into them.

Each state variable $x_{j}^{i}$ represents the normalized particle density and $G_{i}$ gives non-negative output. Therefore, the state space of the network is


(2.7) $$\Omega =[0,1{]}^{n_{1}}\times [0,1{]}^{n_{2}}\ldots [0,1{]}^{n_{N}}\times [0,\infty {)}^{M}.$$

Let


(2.8) $$F(t):=\sum_{j=1}^{M}z_{j}(t)+\sum_{i=1}^{N}\sum_{j=1}^{n_{i}}x_{j}^{i}(t)$$

describe the total occupancy of particles in the network at any time t . Since it is a closed system, F(t) is the first integral of the dynamics. Note that all the particles can be accommodated in any of the pools.

Analysing such networks requires information about interconnected RFMs and the pools, and therefore, a minimal model of a two-pool network provides a useful starting point for studying the behaviour of particle flow in a network with multiple pools. We now shall begin our study with a new model RFMNTP that considers two dynamic pools in the network. The proposed model considers the dynamics of particles on various tracks, wherein on some tracks, particles are recruited from one pool and return to the other pool and vice versa. This is a primary study of a system having two pools in the framework of a network of RFMs. The findings can be generalized to complex systems with multiple pools.

## 3. The ribosome flow model network with two pools

**Figure 3.**
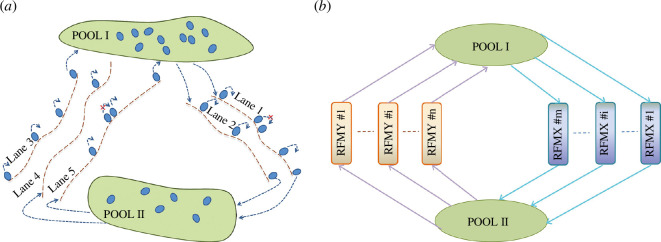
(***a***) Topology of the network with two pools and five lanes: particles from Pool I (Pool II) traverse lanes 1,2 (3,4,5) and join the Pool II (Pool I) and then traverse lanes 3,4,5 (1,2) and again join Pool I (Pool II). Hence, Pool I (Pool II) supplies its input to lanes 1,2 (3,4,5) and receives its output from lanes 3,4,5 (1,2). In the case of vehicular traffic, particle/lane/pool represents the car/road/city. (***b***)Topology of the RFMNTP: the m RFMXs receive their input from Pool I and supply their output to Pool II and the n RFMYs receive their input from Pool II and supply their output to Pool I.

We consider a network model consisting of several RFMs and two finite pools: Pool I and Pool II, and this models the dynamics of the flow of particles on several lanes interconnected via two pools (see figure 3*a*). We represent those RFMs, say m≥1 in number, whose input is received through Pool I, and output is supplied to Pool II as RFMXs. For the reverse case, the RFMs, say n≥1 in number, whose input is received through Pool II, and output is supplied to Pool I are referred to as RFMYs. We call this network RFMNTP (see figure 3*b* ). Let RFMX # i is described by the tuple $E_{i}:=\{\ell_{i},G_{i},x_{j}^{i},\lambda_{k}^{i}\}$ for i=1,2,…,m , $j=1,2\ldots ,\ell_{i}$ , $k=0,1,\ldots ,\ell_{i}$ , where $\ell_{i}$ is the dimension of RFMX # $i;G_{i}:R_{+}⟶R_{+}$ is the i th input function; $x_{j}^{i}$ s are the state variables, and $\lambda_{k}^{i}$ s are the positive transition rates along RFMX # i . Similarly, consider RFMY # i represented by the tuple $F_{i}:=\{p_{i},H_{i},y_{j}^{i},\eta_{k}^{i}\}$ for i=1,2,…,n , $j=1,2\ldots ,p_{i}$ , $k=0,1,\ldots ,p_{i}$ , where $p_{i}$ is the dimension of RFMY # $i;H_{i}:R_{+}⟶R_{+}$ is the i th input function, $y_{j}^{i}$ s are the state variables and $\eta_{k}^{i}$ s are the positive transition rates along RFMY # i . The Pool I and Pool II densities at time t are modelled by $z_{1}(t)$ and $z_{2}(t)$ , respectively.

Thus, RFMX # i dynamics is described by the following equations:


(3.1) $$\begin{array}{rl} {\dot{x}}_{1}^{i} & =\lambda_{0}^{i}G_{i}(z_{1})(1-x_{1}^{i})-\lambda_{1}^{i}x_{1}^{i}(1-x_{2}^{i}),\\ {\dot{x}}_{2}^{i} & =\lambda_{1}^{i}x_{1}^{i}(1-x_{2}^{i})-\lambda_{2}^{i}x_{2}^{i}(1-x_{3}^{i}),\\ & \vdots \\ {\dot{x}}_{\ell_{i}}^{i} & =\lambda_{\ell_{i}-1}^{i}x_{\ell_{i}-1}^{i}(1-x_{\ell_{i}}^{i})-\lambda_{\ell_{i}}^{i}x_{\ell_{i}}^{i}, \end{array}$$

and its output rate of exit of particles is given by $\lambda_{\ell_{i}}^{i}x_{\ell_{i}}^{i}$ .

The dynamics of RFMY # i is described by the following equations:


(3.2) $$\begin{array}{rl} {\dot{y}}_{1}^{i} & =\eta_{0}^{i}H_{i}(z_{2})(1-y_{1}^{i})-\eta_{1}^{i}y_{1}^{i}(1-y_{2}^{i}),\\ {\dot{y}}_{2}^{i} & =\eta_{1}^{i}y_{1}^{i}(1-y_{2}^{i})-\eta_{2}^{i}y_{2}^{i}(1-y_{3}^{i}),\\ & \vdots \\ {\dot{y}}_{p_{i}}^{i} & =\eta_{p_{i}-1}^{i}y_{p_{i}-1}^{i}(1-y_{p_{i}}^{i})-\eta_{p_{i}}^{i}y_{p_{i}}^{i}, \end{array}$$

and its output rate of exit of particles is given by $\eta_{p_{i}}^{i}y_{p_{i}}^{i}$ .

Pool I feeds all the RFMXs, and the output of each RFMY is supplied into Pool I, so the change in $z_{1}$ is given by the following balance equation:


(3.3) $${\dot{z}}_{1}=\sum_{i=1}^{n}\eta_{p_{i}}^{i}y_{p_{i}}^{i}-\sum_{i=1}^{m}\lambda_{0}^{i}\;G_{i}(z_{1})(1-x_{1}^{i}).$$

In addition, Pool II feeds all the RFMYs, and the output of each RFMX is supplied into Pool II, so the change in $z_{2}$ is given by the following balance equation:


(3.4) $${\dot{z}}_{2}=\sum_{i=1}^{m}\lambda_{\ell_{i}}^{i}x_{\ell_{i}}^{i}-\sum_{i=1}^{n}\eta_{0}^{i}\;H_{i}(z_{2})(1-y_{1}^{i}).$$

It can be observed that if the pools are empty, then no particles can attach to the respective lanes, and as the pools become fuller, more particles can attach to the lanes. Therefore, these properties are satisfied by imposing the following assumptions on $G_{i}$ and $H_{i}$ : (i) $G_{i}(0)=0$ and $H_{i}(0)=0$ and (ii) $G_{i}(z_{1})$ and $H_{i}(z_{2})$ are continuous and strictly increasing functions of $z_{1}$ and $z_{2}$ , respectively. Let


(3.5) $$Q(t):=z_{1}(t)+z_{2}(t)\sum_{i=1}^{m}\sum_{j=1}^{\ell_{i}}x_{j}^{i}(t)+\sum_{i=1}^{n}\sum_{j=1}^{p_{i}}y_{j}^{i}(t)$$

describe the total occupancy of particles in the network at any time *t*. Since the RFMNTP is a closed system, Q(t) is the first integral of the dynamics, that is, Q(t)=Q(0) for all t≥0 . Note that both pool densities are bounded by Q(0) . Summing up, the RFMNTP is a dynamical system with $s=\sum_{i=1}^{m}\ell_{i}\;+\;\sum_{i=1}^{n}p_{i}+2$ state variables whose dynamics are given by equations (3.1)–(3.4). The next section rigorously analyses the mathematical aspect of the RFMNTP.

### 3.1. Dynamical properties of the ribosome flow model network with two pools

In this section, we shall describe the various dynamical properties related to the RFMNTP. Given two vectors u , $v\in R^{n}$ , we define order relation u≪v if $u_{i}<v_{i}$ for all i . Recall that every $x_{j}^{i}$ and $y_{j}^{i}$ represent normalized particle density, and the assumptions on $G_{i}$ and $H_{i}$ imply that the pool densities are always non-negative. Therefore, the state space of the RFMNTP is


(3.6) $$B=[0,1{]}^{\ell_{1}}\times [0,1{]}^{\ell_{2}}\times \ldots [0,1{]}^{\ell_{m}}\times [0,1{]}^{p_{1}}\times [0,1{]}^{p_{2}}\times \ldots [0,1{]}^{p_{n}}\times [0,\infty )\times [0,\infty ).$$

Let $[x(t,a)\;y(t,a)\;z_{1}(t,a)\;z_{2}(t,a){]}^{\prime }$ denote the solution of the RFMNTP at time t for the initial condition a∈B , where x and y are the vector consisting of all the state variables of RFMXs and RFMYs, respectively. For r≥0 , let $L_{r}:=\{a\in B:\sum_{i=1}^{s}a_{i}=r\}$ , that is, $L_{r}$ represents all states in B corresponding to the total occupancy of particles equal to r in the network. In other words, $L_{r}$ is the r level set of first integral Q .

### 3.2. Invariance

The following result states that for any initial condition a∈B , the trajectory of the RFMNTP stays in B for all t≥0 and follows by analysing the equations of the RFMNTP.

**Proposition 3.1** *The state space* B *is an invariant set of the RFMNTP, that is,* $0\leq x_{j}^{i}(t,a)\leq 1$ , $0\leq y_{j}^{i}(t,a)\leq 1$ , $z_{i}(t,a)\in [0,\infty )$ *for any* t≥0 *and any initial condition* a∈B .

We shall now show that the proposed nonlinear system of differential equations is a cooperative system (all the non-diagonal entries of the Jacobian matrix are non-negative in the invariant state space B ). This property guarantees the monotonicity (order-preserving property) of the flow with respect to the partial ordering in the phase space [34]. Now, the Jacobian matrix J of the vector field of the RFMNTP is


$$\begin{array}{r} J(x,y,z_{1},z_{2})=\left[\begin{array}{cccccccccc} X_{1} & 0 & 0 & 0 & 0 & 0 & \ldots & 0 & U_{1} & 0\\ 0 & X_{2} & 0 & 0 & 0 & 0 & \ldots & 0 & U_{2} & 0\\ \vdots & & \ddots & \vdots & \vdots & \vdots & & \vdots & \vdots & \vdots \\ 0 & 0 & \ldots & X_{m} & 0 & 0 & \ldots & 0 & U_{m} & 0\\ 0 & 0 & \ldots & 0 & Y_{1} & 0 & \ldots & 0 & 0 & V_{1}\\ 0 & 0 & \ldots & 0 & 0 & Y_{2} & \ldots & 0 & 0 & V_{2}\\ \vdots & \vdots & & \vdots & \vdots & & \ddots & \vdots & \vdots & \vdots \\ 0 & 0 & \ldots & 0 & 0 & 0 & \ldots & Y_{n} & 0 & V_{n}\\ A_{1} & A_{2} & \ldots & A_{m} & B_{1} & B_{2} & \ldots & B_{n} & Z_{1} & 0\\ C_{1} & C_{2} & \ldots & C_{m} & D_{1} & D_{2} & \ldots & D_{n} & 0 & Z_{2} \end{array}\right] \end{array},$$

where $X_{i}$ represents the Jacobian matrix of RFMX #i and is given by


$$\begin{array}{r} \left[\begin{array}{ccccc} -\lambda_{0}^{i}G_{i}(z_{1})-\lambda_{1}^{i}(1-x_{2}^{i}) & \lambda_{1}^{i}x_{1}^{i} & 0 & 0 & 0\\ \lambda_{1}^{i}(1-x_{2}^{i}) & -\lambda_{1}^{i}x_{1}^{i}-\lambda_{2}^{i}(1-x_{3}^{i}) & \lambda_{2}^{i}x_{2}^{i} & 0 & 0\\ & & \ddots \\ 0 & 0 & 0 & -\lambda_{\ell_{i}-2}^{i}x_{\ell_{i}-2}^{i}-\lambda_{\ell_{i}-1}^{i}(1-x_{\ell_{i}}^{i}) & \lambda_{\ell_{i}-1}^{i}x_{\ell_{i}-1}^{i}\\ 0 & 0 & 0 & \lambda_{\ell_{i}-1}^{i}(1-x_{\ell_{i}}^{i}) & -\lambda_{\ell_{i}-1}^{i}x_{\ell_{i}-1}^{i}-\lambda_{\ell_{i}}^{i} \end{array}\right], \end{array}$$

and $Y_{i}$ represents the Jacobian matrix of RFMY #i and is given by


$$\begin{array}{r} \left[\begin{array}{ccccc} -\eta_{0}^{i}H_{i}(z_{2})-\eta_{1}^{i}(1-y_{2}^{i}) & \eta_{1}^{i}y_{1}^{i} & 0 & 0 & 0\\ \eta_{1}^{i}(1-y_{2}^{i}) & -\eta_{1}^{i}y_{1}^{i}-\eta_{2}^{i}(1-y_{3}^{i}) & \eta_{2}^{i}y_{2}^{i} & 0 & 0\\ & & \ddots \\ 0 & 0 & 0 & -\eta_{p_{i}-2}^{i}y_{p_{i}-2}^{i}-\eta_{p_{i}-1}^{i}(1-y_{p_{i}}^{i}) & \eta_{p_{i}-1}^{i}y_{p_{i}-1}^{i}\\ 0 & 0 & 0 & \eta_{p_{i}-1}^{i}(1-y_{p_{i}}^{i}) & -\eta_{p_{i}-1}^{i}y_{p_{i}-1}^{i}-\eta_{p_{i}}^{i} \end{array}\right], \end{array}$$

$A_{i}=[\lambda_{0}^{i}G_{i}(z_{1})\;0\ldots 0\;0]$ , $B_{i}=[0\ldots 0\;0\;\eta_{p_{i}}^{i}]$ , $C_{i}=[0\ldots 0\;0\;\lambda_{\ell_{i}}^{i}]$ , $D_{i}=[\eta_{0}^{i}H_{i}(z_{2})\;0\ldots 0\;\;0]$ , $U_{i}=[\lambda_{0}^{i}G_{i}^{\prime }(z_{1})(1-x_{1}^{i})\;0...\;0{]}^{\prime }$ , $V_{i}=[\eta_{0}^{i}H_{i}^{\prime }(z_{2})(1-y_{1}^{i})\;0\ldots 0{]}^{\prime }$ , $Z_{1}=-\sum_{i=1}^{m}\lambda_{0}^{i}G_{i}^{\prime }(z_{1})(1-x_{1}^{i})$ and $Z_{2}=-\sum_{i=1}^{n}\eta_{0}^{i}H_{i}^{\prime }(z_{2})(1-y_{1}^{i})$ . Clearly, by proposition 3.1, we get that the Jacobian matrix J is Metzler for any initial condition in B , and thus, the RFMNTP is a cooperative dynamical system.

### 3.3. Persistence

The next result proves that the property called persistence holds, which implies that any trajectory becomes uniformly separated from the boundary of B .

**Proposition 3.2** *For any* δ>0 *there exists* ϵ>0 *depending on* δ *with* ϵ→0 *as* δ→0 *such that* $\epsilon \leq x_{j}^{i}(t,a)\leq 1-\epsilon$ *, for* i∈{1,2,…,m} *,* $j\in \{1,2,\ldots ,\ell_{i}\}$ *,* $\epsilon \leq y_{j}^{i}(t,a)\leq 1-\epsilon$ *, for* i∈{1,2,…,n} *,* $j\in \{1,2,\ldots ,p_{i}\}$ *and* $\epsilon \leq z_{i}(t,a)$ *for* i∈{1,2} *for all* a∈(B∖{0}) *and all* t≥δ *.*

In other words, all the densities are smaller than one and larger than zero, and the pool occupancies are strictly positive. This result guarantees that the Jacobian matrix of the dynamics becomes irreducible after an arbitrarily short time [56]. Thus, the RFMNTP is a cooperative irreducible system of ODEs. Next, we analyse the asymptotic behaviour of the RFMNTP.

### 3.4. Stability

The following result shows that each level set of the first integral has a unique intersection with the ordered set of fixed points.

**Theorem 3.1** *The RFMNTP admits a unique steady-state point in every level set* $L_{r}$ *, say* $e_{r}$ *, and for any initial condition* $a\in L_{r}$ *, the trajectory converges to* $e_{r}$ *. Furthermore, for any* $0\leq r_{1}<r_{2}$ *, we have* $e_{r_{1}}\ll e_{r_{2}}$ *.*

The above theorem implies that the rates $\lambda_{j}^{i}$ and $\eta_{j}^{i}$ , and the total density of particle r determine a unique steady-state point of the network. Moreover, the continuum of the steady-state points $\left\{e_{r}:r\in [0,\infty )\right\}$ is linearly ordered. Combining proposition 3.2 and theorem 3.1, it follows that for any r>0 , $e_{r}\in \text{Int}(B)$ , that is, the steady-state profile will never include densities of RFMXs and RFMYs that are either zero or one and the Pool I, and the Pool II steady-state densities are always strictly positive. The following example demonstrates theorem 3.1.

**Figure 4.**
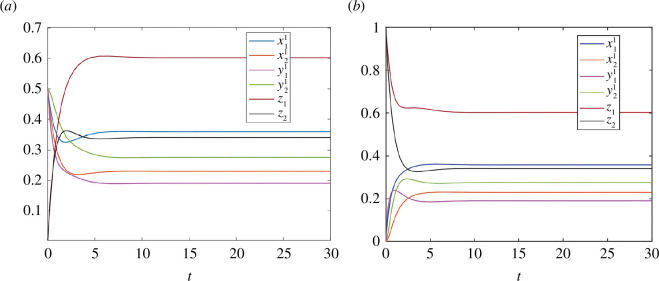
Trajectories of the RFMNTP in example 3.1: (***a***) for initial condition, $[0.5\;0.5\;0.5\;0.5\;0\;0{]}^{\prime }$ and (***b***) for initial condition, $[0\;0\;0\;0\;1\;1{]}^{\prime }$ .

**Example 3.1** Consider an RFMNTP with m=1 RFMX and n=1 RFMY each with dimension 2 . Assume that $\lambda_{0}^{1}=0.8$ , $\lambda_{1}^{1}=1$ , $\lambda_{2}^{1}=1.2$ , $\eta_{0}^{1}=1$ , $\eta_{1}^{1}=2$ , $\eta_{2}^{1}=1$ , $G_{1}(z_{1})=\tanh(z_{1})$ and $H_{1}(z_{2})=z_{2}$ . By theorem 3.1, there exists a unique equilibrium point e in $L_{2}$ , and after simulating the dynamical system, we have $e=[0.3589\;0.2302\;0.1909\;0.2763\;0.6023\;0.3414{]}^{\prime }$ . Figure 4*a*,*b* depicts trajectories of RFMNTP for initial conditions in the level set $L_{2}:[0.5\;0.5\;0.5\;0.5\;0\;0{]}^{\prime }$ and $[0\;0\;0\;0\;1\;1{]}^{\prime }$ , respectively. It can be observed that each of these trajectories converges to e .

For ease of notation, let $e=[e_{x}\;e_{y}\;e_{z_{1}}\;e_{z_{2}}{]}^{\prime }$ where $e_{x}:=[e_{x_{1}}^{1}\;e_{x_{2}}^{1}\cdots e_{x_{\ell_{1}}}^{1}e_{x_{1}}^{2}e_{x_{2}}^{2}\cdots e_{x_{\ell_{2}}}^{2}\cdots e_{x_{1}}^{m}e_{x_{2}}^{m}\cdots e_{x_{\ell_{m}}}^{m}]$ and $e_{y}:=[e_{y_{1}}^{1}e_{y_{2}}^{1}\cdots e_{y_{p_{1}}}^{1}e_{y_{1}}^{2}e_{y_{2}}^{2}\cdots e_{y_{p_{2}}}^{2}\cdots e_{y_{p_{1}}}^{n}e_{y_{p_{2}}}^{n}\cdots e_{y_{p_{n}}}^{n}]$ denote the unique steady-state point of the RFMNTP in the level set $L_{r}$ of Q . At steady-state, the change in all the state variables and the pool variables with respect to time t becomes zero and thereby equation (3.1) implies


(3.7) $$\lambda_{0}^{i}G_{i}(e_{z_{1}})(1-e_{x_{1}}^{i})=\lambda_{1}^{i}e_{x_{1}}^{i}(1-e_{x_{2}}^{i})=\cdots =\lambda_{\ell_{i}}^{i}e_{x_{\ell_{i}}}^{i}.$$

Similarly, at steady state, equation (3.2) yields


(3.8) $$\eta_{0}^{i}H_{i}(e_{z_{2}})(1-e_{y_{1}}^{i})=\eta_{1}^{i}e_{y_{1}}^{i}(1-e_{y_{2}}^{i})=\cdots =\eta_{p_{i}}^{i}e_{y_{p_{i}}}^{i}.$$

Again at steady state, equation (3.3) implies that


(3.9) $$\sum_{i=1}^{n}\eta_{p_{i}}^{i}e_{y_{p_{i}}}^{i}=\sum_{i=1}^{m}\lambda_{0}^{i}\;G_{i}(e_{z_{1}})(1-e_{x_{1}}^{i}).$$

We can also express the above equation as


(3.10) $$\sum_{i=1}^{n}\eta_{p_{i}}^{i}e_{y_{p_{i}}}^{i}=\sum_{i=1}^{m}\lambda_{\ell_{i}}^{i}e_{x_{\ell_{i}}}^{i}.$$

Equation (3.10) implies that the total output of all the RFMXs is equal to the total output of all the RFMYs.

Next, we provide an analysis of how the spectral approach can obtain the steady-state e of the network without any numerical simulations of the dynamics. Consider an RFMNTP with m RFMXs where each RFMX has dimension $\ell_{i}$ , i=1,2,…,m and n RFMYs, where each RFMY has dimension $p_{k}$ , k=1,2,…,n . We also assume that the transition rates in RFMX # i are represented by $\lambda_{j}^{i}$ and in RFMY # k by $\eta_{k}^{i}$ . The input to RFMX # i is given by $G_{i}$ and to RFMY # k by $H_{k}$ . Consider the total density of particles in the network to be r .

The steady-state values of each RFMX # i satisfy


(3.11) $$e_{x_{j}}^{i}=\frac{\zeta_{j+2}^{i}}{\sqrt{\lambda_{j}^{i}}\sigma_{x}^{i}\zeta_{j+1}^{i}},\;\;j=1,2,\ldots ,\ell_{i},$$

where $\sigma_{x}^{i}$ is the Perron eigenvalue and $\zeta^{i}$ is the corresponding Perron eigenvector of the $\left(\ell_{i}+2)\times (\ell_{i}+2\right)$ Jacobi matrix $A_{i}$ given by


$$A_{i}:=\left[\begin{array}{ccccc} 0 & (\lambda_{0}^{i}G_{i}(e_{z_{1}}){)}^{-1/2} & 0 & \ldots & 0\\ (\lambda_{0}^{i}G_{i}(e_{z_{1}}){)}^{-1/2} & 0 & (\lambda_{1}^{i}{)}^{-1/2} & \ldots & 0\\ 0 & (\lambda_{1}^{i}{)}^{-1/2} & 0 & \ldots & 0\\ & & \vdots \\ 0 & \ldots & 0 & 0 & (\lambda_{\ell_{i}}^{i}{)}^{-1/2}\\ 0 & \ldots & 0 & (\lambda_{\ell_{i}}^{i}{)}^{-1/2} & 0 \end{array}\right].$$

The steady-state values of each RFMY # k satisfy


(3.12) $$e_{y_{j}}^{k}=\frac{\xi_{j+2}^{k}}{\sqrt{\eta_{j}^{k}}\sigma_{y}^{k}\xi_{j+1}^{k}},\;\;j=1,2,\ldots ,p_{k},$$

where $\sigma_{y}^{k}$ is the Perron eigenvalue and $\xi^{k}$ is the corresponding Perron eigenvector of the $\left(p_{k}+2)\times (p_{k}+2\right)$ Jacobi matrix $B_{k}$ given by


$$B_{k}:=\left[\begin{array}{ccccc} 0 & (\eta_{0}^{k}H_{k}(e_{z_{2}}){)}^{-1/2} & 0 & \ldots & 0\\ (\eta_{0}^{k}H_{k}(e_{z_{2}}){)}^{-1/2} & 0 & (\eta_{1}^{k}{)}^{-1/2} & \ldots & 0\\ 0 & (\eta_{1}^{k}{)}^{-1/2} & 0 & \ldots & 0\\ & & \vdots \\ 0 & \ldots & 0 & 0 & (\eta_{p_{k}}^{k}{)}^{-1/2}\\ 0 & \ldots & 0 & (\eta_{p_{k}}^{k}{)}^{-1/2} & 0 \end{array}\right].$$

It follows from equation (3.5) that


(3.13) $$e_{z_{1}}+e_{z_{2}}+\sum_{i=1}^{m}\sum_{j=1}^{\ell_{i}}e_{x_{j}}^{i}(e_{z_{1}})+\sum_{k=1}^{n}\sum_{j=1}^{p_{k}}e_{y_{j}}^{k}(e_{z_{2}})=r.$$

Also, equation (3.10) implies


(3.14) $$\sum_{i=1}^{n}\eta_{p_{i}}^{i}e_{y_{p_{i}}}^{i}(e_{z_{2}})=\sum_{i=1}^{m}\lambda_{\ell_{i}}^{i}e_{x_{\ell_{i}}}^{i}(e_{z_{1}}).$$

Combining equations (3.13) and (3.14) gives the expression of $e_{z_{1}}$ and $e_{z_{2}}$ in terms of the number of particles r . Thus, the entire steady-state profile of the network can then be calculated by plugging the values of r in the expression. Note that this approach also allows one to obtain an expression of densities for any unknown transition parameter and we only need to plug the values to obtain the entire steady-state profile without any numerical simulations of the dynamics. However, one needs to choose stable algorithms to solve the system of nonlinear equations (3.13) and (3.14).

### 3.5. Entrainment

Many important dynamic processes are periodic, such as the cell-cycle division process, gene regulation, circadian rhythm, 24 h solar day and more [58–60]. Proper functioning often requires such processes to vary periodically within the same period. For example, a person’s lack of synchronization between day and night can have health consequences [61]. For nonlinear systems, a periodic input signal does not guarantee that the response of the system will also be periodic as their behaviour can be quasi-periodic or chaotic [62,63]. Therefore, a natural question is whether the RFMNTP synchronizes with the periodic excitations or not. To answer this question, we assume that some or all of the parameters in the RFMNTP are not constants but periodic and continuous functions of time with a common period T>0 and satisfy the condition $0<\delta_{1}<\lambda_{j}^{i}\leq \delta_{2}$ and $0<\delta_{3}<\eta_{j}^{i}\leq \delta_{4}$ . In this case, we call the network model the *periodic* RFMNTP (PRFMNTP). The next result shows that all the trajectories approach a periodic pattern with the same period T .

**Theorem 3.2.** *Consider the PRFMNTP. Fix* r>0 *. Then a unique* T *-periodic function* $\phi_{r}:R_{+}⟶\text{Int}(B)$ *exists and for any* $a\in L_{r}$ *, the solution of the PRFMNTP converges to* $\phi_{r}$ *.*

In particular, PRFMNTP entrains to the periodic excitations in the parameters. As an additional point, if we examine the RFMNTP model for vehicular traffic between two cities, it enables a continuous flow of traffic while coordinating with the traffic lights. In simpler terms, when the traffic lights (rates) change periodically, the traffic density (state variables) will gradually settle into a recurring pattern within the same period. The following example illustrates the dynamic behaviour of the PRFMNTP model.

**Figure 5.**
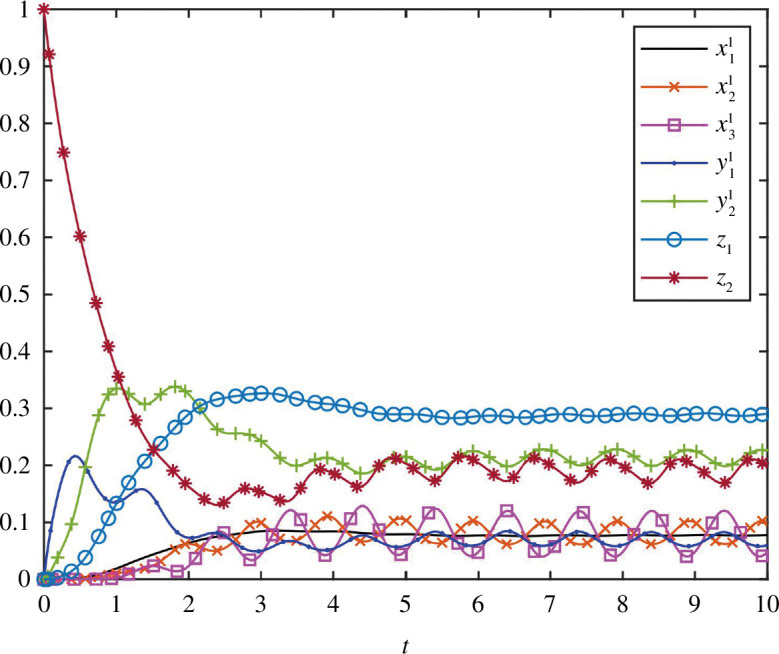
Trajectories of the PRFMNTP in example 3.2 as a function of time. Each state variable converges to a periodic solution having period one.

**Example 3.2.** Consider a PRFMNTP with m=1 RFMX with dimension $\ell_{1}=3$ and n=1 RFMY with dimensions $p_{1}=2$ . Assume that $\lambda_{0}^{1}=0.8$ , $\lambda_{1}^{1}=3$ , $\lambda_{2}^{1}=3+2\sin(2\pi t)$ , $\lambda_{3}^{1}=3-2\sin(2\pi t)$ , $\eta_{0}^{1}=1.2$ , $\eta_{1}^{1}=4-2\sin(2\pi t)$ , $\eta_{2}^{1}=1$ , $G_{1}(z_{1})=z_{1}$ and $H_{1}(z_{2})=z_{2}$ . Let initial condition be $x_{j}^{i}=0$ , $y_{j}^{i}=0$ , $z_{1}(0)=0$ and $z_{2}(0)=1$ . Note that all the parameters are periodic with a common period T=1 . It can be seen in figure 5 that every trajectory converges to a periodic function with period one.

### 3.6. Effect of parameters

In this subsection, we analyse the effect of change in parameters on the network using a theoretical framework that is well explained through simple examples. Without loss of generality, we analyse the effect of change in the transition rate at a single site of an RFMX on the steady-state point of the network and presume that the change is in one of the transition rates in RFMX #1 . Let $\lambda :=[\lambda_{0}^{1}\cdots \lambda_{\ell_{1}}^{1}\;\lambda_{0}^{2}\cdots \lambda_{\ell_{2}}^{2}\cdots \lambda_{0}^{m}\cdots \lambda_{\ell_{m}}^{m}]$ and $\eta :=[\eta_{0}^{1}\cdots \eta_{p_{1}}^{1}\eta_{0}^{2}\cdots \eta_{p_{2}}^{2}\cdots \eta_{0}^{n}\cdots \eta_{p_{n}}^{n}]$ .

**Theorem 3.3.** *Consider an RFMNTP with* m *RFMXs having dimensions* $\ell_{1},\ell_{2},\ldots ,\ell_{n}$ *and* n *RFMYs having dimensions* $p_{1},p_{2},\ldots ,p_{n}$ *. Let* $P=[\lambda \;\;\eta {]}^{\prime }$ *denotes the set of all parameters of the RFMNTP. Fix* r>0 *and let* e *denotes the unique steady-state point of the RFMNTP in the level set* $L_{r}$ *of* Q *. Pick* $k\in \{0,1,\ldots ,\ell_{1}\}$ *and suppose that we modify* $\lambda_{k}^{1}$ *to* ${\bar{\lambda }}_{k}^{1}$ *with* $\lambda_{k}^{1}<{\bar{\lambda }}_{k}^{1}$ *. Let* $\bar{e}$ *denotes the steady-state point in the new RFMNTP. Then*


(3.15) $${\bar{e}}_{x_{k}}^{1}<e_{x_{k}}^{1}\;\;\text{and}\;\;e_{x_{j}}^{1}<{\bar{e}}_{x_{j}}^{1}\;\;\text{for all}\;\;j\in \{k+1,\ldots ,\ell_{1}\}.$$

Also, *either one of the following cases holds*:

1.
$e_{z_{1}}<{\bar{e}}_{z_{1}}$ *,* $e_{z_{2}}<{\bar{e}}_{z_{2}}$ *,* $e_{x_{j}}^{i}<{\bar{e}}_{x_{j}}^{i}$ *for all* i∈{2,3,…,m} *,* $j\in \{1,2,\ldots ,\ell_{i}\}$ *and* $e_{y_{j}}^{i}<{\bar{e}}_{y_{j}}^{i}$ *for all* i∈{1,2,…,n} *,* $j\in \{1,2,\ldots ,p_{i}\}$ .
2.
${\bar{e}}_{z_{1}}<e_{z_{1}}$ *,* $e_{z_{2}}<{\bar{e}}_{z_{2}}$ *,* ${\bar{e}}_{x_{j}}^{i}<e_{x_{j}}^{i}$ *for all* i∈{2,3,…,m} *,* $j\in \{1,2,\ldots ,\ell_{i}\}$ *and* $e_{y_{j}}^{i}<{\bar{e}}_{y_{j}}^{i}$ *for all* i∈{1,2,…,n} *,* $j\in \{1,2,\ldots ,p_{i}\}$ .
3.
${\bar{e}}_{z_{1}}<e_{z_{1}}$ *,* ${\bar{e}}_{z_{2}}<e_{z_{2}}$ *,* ${\bar{e}}_{x_{j}}^{i}<e_{x_{j}}^{i}$ *for all* i∈{2,3,…,m} *,* $j\in \{1,2,\ldots ,\ell_{i}\}$ *and* ${\bar{e}}_{y_{j}}^{i}<e_{y_{j}}^{i}$ *for all* i∈{1,2,…,n} *,* $j\in \{1,2,\ldots ,p_{i}\}$ .
4.
$e_{z_{1}}={\bar{e}}_{z_{1}}$ *,* $e_{z_{2}}<{\bar{e}}_{z_{2}}$ *,* $e_{x_{j}}^{i}={\bar{e}}_{x_{j}}^{i}$ *for all* i∈{2,3,…,m} *,* $j\in \{1,2,\ldots ,\ell_{i}\}$ *and* $e_{y_{j}}^{i}<{\bar{e}}_{y_{j}}^{i}$ *for all* i∈{1,2,…,n} *,* $j\in \{1,2,\ldots ,p_{i}\}$ .
5.
${\bar{e}}_{z_{1}}<e_{z_{1}}$ *,* $e_{z_{2}}={\bar{e}}_{z_{2}}$ *,* ${\bar{e}}_{x_{j}}^{i}<e_{x_{j}}^{i}$ *for all* i∈{2,3,…,m} *,* $j\in \{1,2,\ldots ,\ell_{i}\}$ *and* $e_{y_{j}}^{i}={\bar{e}}_{y_{j}}^{i}$ *for all* i∈{1,2,…,n} *,* $j\in \{1,2,\ldots ,p_{i}\}$ .

Clearly, the above theorem lists all the possible cases of the effect of modifying the transition rate $\lambda_{k}^{1}$ on the steady-state densities of the remaining RFMXs, all the RFMYs, and the pools. However, it does not give any information on the modified steady-state densities in sites {1,…,k−1} of RFMX #1 . Note that the theorem also exhibits how the output rates in all the RFMXs and RFMYs change. The next example demonstrates that the scenario when modifying a slow site increases the output rates of all lanes.

**Figure 6.**
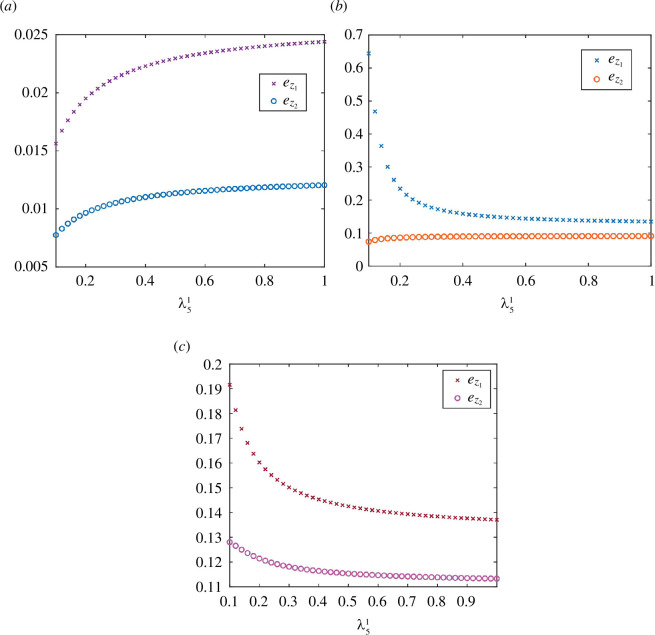
The steady-state pool densities for various values of transition rate $\lambda_{5}^{1}$ in RFMX #1 of the RFMNTP considered in (***a***) example 3.3, (***b***) example 3.4 and (***c***) example 3.5.

**Example 3.3.** Consider an RFMNTP with m=1 RFMX with dimension $\ell_{1}=10$ and n=2 RFMYs with dimensions $p_{i}=5$ for i=1,2 . Assume that $\lambda_{j}^{1}=1$ for i=1 , $j=1,2,\ldots ,\ell_{i}$ , $\eta_{j}^{i}=1$ for i=1,2 and $j=1,2,\ldots ,p_{i}$ , $G_{1}(z_{1})=z_{1}$ and $H_{i}(z_{2})=z_{2}$ for i=1,2 . Let initial point be $x_{j}^{i}=0$ , $y_{j}^{i}=0$ , $z_{1}(0)=0.2$ and $z_{2}(0)=0.2$ . We simulate the system until steady state for a range of values of $\lambda_{5}^{1}$ . It can be seen in figure 6*a* that we have $e_{z_{1}}<{\bar{e}}_{z_{1}}$ and $e_{z_{2}}<{\bar{e}}_{z_{2}}$ . Note that when $\lambda_{5}^{1}$ is small, it is the only bottleneck rate in the RFMX and increasing it allows more particles to traverse RFMX more quickly. Hence, this increases the output flow rate of the RFMX, and subsequently, the Pool II density increases. This further increases the output rates of all the RFMYs and thus increases the Pool I density.

It has been previously reported that owing to environmental change, stress conditions or pathological conditions, there could be particle stalling leading to an increase in a traffic jam in a track, resulting in a decrement in output rates of other tracks [25]. However, in our model, resource sharing is based on the concept that the entry rate is impacted by the neighbouring particles, and this can lead to both effects: a decrease in output rates from some of the tracks and an increase in output rates from others. The next example demonstrates this.

**Example 3.4.** Consider an RFMNTP with m=1 RFMX with dimension $\ell_{1}=20$ and n=1 RFMY with dimension $p_{1}=10$ . Assume that $\lambda_{j}^{1}=1$ for i=1 , $j=1,2,\ldots ,\ell_{i}$ except $\lambda_{7}^{1}=0.1$ , $\eta_{j}^{i}=1$ for i=1 and $j=1,2,\ldots ,p_{i}$ , $G_{1}(z_{1})=z_{1}$ and $H_{1}(z_{2})=z_{2}$ . Consider an initial condition $x_{j}^{i}=0$ , $y_{j}^{i}=0$ , $z_{1}(0)=4$ and $z_{2}(0)=4$ . We simulate the system until steady state for a range of values of $\lambda_{5}^{1}$ . It can be seen in figure 6*b* that we have ${\bar{e}}_{z_{1}}<e_{z_{1}}$ and $e_{z_{2}}<{\bar{e}}_{z_{2}}$ . This can be explained as follows. Note that when $\lambda_{7}^{1}$ is the bottleneck rate in the RFMX, increasing $\lambda_{5}^{1}$ only generates more traffic jams along RFMX. This depletes the Pool I density. However, in this case, the number of particles increases on the RFMX, and thus, the output flow rate of the RFMX increases, and subsequently, the Pool II density increases.

The following example exhibits the scenario in which increasing any of the transition rates in a specific lane yields an increase in the output rate of this lane, and the output rates in the other lanes all decrease.

**Example 3.5.** Consider an RFMNTP with m=2 RFMX with dimensions $\ell_{1}=10$ , $\ell_{2}=5$ and n=2 RFMYs with dimensions $p_{i}=5$ for i=1,2 . Assume that $\lambda_{j}^{i}=1$ for i=1,2 , $j=1,2,\ldots ,\ell_{i}$ , $\eta_{j}^{i}=1$ for i=1,2 and $j=1,2,\ldots ,p_{i}$ , $G_{i}(z_{1})=z_{1}$ and $H_{i}(z_{2})=z_{2}$ for i=1,2 . Consider an initial condition $x_{j}^{i}=0$ , $y_{j}^{i}=0$ , $z_{1}(0)=4$ and $z_{2}(0)=4$ . We simulate the system until steady state for a range of values of $\lambda_{5}^{1}$ . It can be seen in figure 6*c* that we have ${\bar{e}}_{z_{1}}<e_{z_{1}}$ and ${\bar{e}}_{z_{2}}<e_{z_{2}}$ . This can be understood by the following explanation. Increasing $\lambda_{5}^{1}$ leads to the formation of traffic jams along RFMX # 1 owing to the bottleneck rate $\lambda_{7}^{1}$ . This depletes Pool I and decreases the output rate of RFMX # 2 . So, there is a trade-off between the output values of both RFMXs, that is, whether the rate of increment of the output of RFMX # 1 is higher than the rate of decrement of RFMX # 2 . Depending upon the parameters of the RFMNTP, it can be seen in figure 6*c* that Pool II density decreased owing to the overall decrease in the total output rates from both RFMXs.

The next result provides specific information for the case when there is a single RFMX in the network.

**Corollary 3.1.** *Consider an RFMNTP with* m=1 *RFMX and* n *RFMYs. Pick* $k\in \{0,1,\ldots ,\ell_{1}\}$ *and suppose that* $\lambda_{k}^{1}$ *is changed to* ${\bar{\lambda }}_{k}^{1}$ *with* $\lambda_{k}^{1}<{\bar{\lambda }}_{k}^{1}$ *. Then,*


(3.16) $${\bar{e}}_{x_{k}}^{1}<e_{x_{k}}^{1}\;\;\text{and}\;\;e_{x_{j}}^{1}<{\bar{e}}_{x_{j}}^{1}\;\;\text{for all}\;\;j\in \{k+1,\ldots ,\ell_{1}\},$$

(3.17) $$e_{z_{2}}<{\bar{e}}_{z_{2}},\;\;\text{and}\;\;e_{y_{j}}^{i}<{\bar{e}}_{y_{j}}^{i}\;\;\text{for all}\;\;i\in \{1,2,\ldots ,n\},j\in \{1,2,\ldots ,p_{i}\}.$$

The following result implies that we can study steady-state properties of a network of m identical RFMXs and m identical RFMYs by a much simpler network consisting of only a single RFMX and a single RFMY.

**Proposition 3.3.** *Consider the following two RFMNTPs:*

*(a) An RFMNTP with* m *identical RFMXs each having length* ℓ *, rates* $\lambda_{0},\lambda_{1}\ldots ,\lambda_{\ell }$ *and* m *identical RFMYs each having length* p *, rates* $\eta_{0},\eta_{1}\ldots ,\eta_{p}$ *. Let the output function* G *of Pool I and* H *of Pool II be homogeneous functions of degree* 1 *. Let* r>0 *be the total density of particles in the network and* e *denote its steady-state point.*

*(b) An RFMNTP with a single RFMX of length* ℓ *, rates* $(m\lambda_{0}),\lambda_{1}\ldots ,\lambda_{\ell }$ *and a single RFMY of length* p *, rates* $(m\eta_{0}),\eta_{1}\ldots ,\eta_{p}$ *. Let the output function* G *of Pool I and* H *of Pool II be homogeneous functions of degree* one*. Let* r/m *be the total density of particles in the network and* $\tilde{e}=[{\tilde{e}}_{x_{1}}{\tilde{e}}_{x_{2}}\cdots {\tilde{e}}_{x_{\ell }}\;{\tilde{e}}_{y_{1}}\;{\tilde{e}}_{y_{2}}\cdots {\tilde{e}}_{y_{p}}\;\;{\tilde{e}}_{z_{1}}\;{\tilde{e}}_{z_{2}}]$ *denotes its steady-state point.*

*Then, we have*


(3.18) $$e_{x_{j}}^{i}={\tilde{e}}_{x_{j}}\;\;\text{for all}\;\;i=1,2,\ldots ,m,j=1,2,\ldots \ell ,$$

(3.19) $$e_{y_{j}}^{i}={\tilde{e}}_{y_{j}}\;\text{for all}\;\;i=1,2,\ldots ,m,j=1,2,\ldots p,$$

and


(3.20) $$e_{z_{1}}=m{\tilde{e}}_{z_{1}}\;\text{and}\;e_{z_{2}}=m{\tilde{e}}_{z_{2}}.$$

### 3.7. Mapping of the RFMNP to RFMNTP

In this section, we show that the RFMNP is a special case of our model RFMNTP. The RFMNP has been used for analysing the competition of ribosomes in the translation process. It assumes that the ribosomes that are located far away will also impact the initiation rates of the mRNAs and, therefore, include several RFMs interconnected via a single pool of free particles. All the RFMs feed the pool and the pool feeds the entry locations in all the RFMs. In this section, we show that the model RFMNP can be studied by the model RFMNTP, that is, we can construct the model RFMNP through RFMNTP as illustrated in the next paragraph.

Consider an RFMNP having m RFMs with dimensions $\ell_{i}$ , rates $\lambda_{j}^{i}$ , state variables $x_{j}^{i}$ , a pool with density z , and the first integral having value (1/2)Q(0) . Construct an RFMNTP having m RFMXs and m RFMYs with the assumption $\ell_{i}=p_{i}$ for all i=1,2,…,m , $\lambda_{j}^{i}=\eta_{j}^{i}$ for all i=1,2,…,m , $j=0,1,\ldots ,\ell_{i}$ , $G_{i}=H_{i}$ for each i=1,2…,m and having the first integral Q(0) . We shall show that both Pool I and Pool II steady-state density value is the same, that is, $e_{z_{1}}=e_{z_{2}}$ . Suppose on the contrary $e_{z_{1}}<e_{z_{2}}$ . Note that $G_{i}$ is well defined and strictly increasing function and this implies $G_{i}(e_{z_{1}})<G_{i}(e_{z_{2}})$ for all *i*. Since each RFMY is a copy of an RFMX, therefore, we have $e_{x_{\ell_{i}}}^{i}<e_{y_{\ell_{i}}}^{i}\;\text{for all}\;i$ , and this implies that $\sum_{i=1}^{m}\lambda_{\ell_{i}}^{i}(e_{y_{\ell_{i}}}^{i}-e_{x_{\ell_{i}}}^{i})>0$ , which is a contradiction to equation (3.10) in our case. Hence, $e_{z_{1}}=e_{z_{2}}$ , which implies that the steady-state densities of each RFMX # i are the same as of each RFMY # i , respectively. Therefore, our system becomes equivalent to the given one-pool network RFMNP.

### 3.8. Monte Carlo simulations

It has been shown that RFM and Monte Carlo simulations (TASEP with parallel update rule) provide highly correlated predictions for a large set of parameters [28]. In this section, we compare the steady states of the RFMNTP with the Monte Carlo simulations. This supports the modelling of the network of RFMs with two pools.

We validate our model by performing Monte Carlo simulations with a parallel update scheme. Each site is occupied with at most one particle, and the particle advances to the consecutive site if it is time to hop. The hopping times between consecutive sites of lanes receiving inputs from Pool I is exponentially distributed with parameters $\lambda_{1}^{i},\lambda_{2}^{i},\ldots ,\lambda_{\ell_{i}}^{i}$ , that is, the next hopping time at site j of lane i is $t+e(\lambda_{j}^{i})$ , where t is the current time and $e(\lambda_{j}^{i})$ is randomly generated from the exponential distribution with mean parameter $\lambda_{j}^{i}$ and the hopping time for the particle to hop to site 1 of lane i is calculated as $t+e(\lambda_{0}^{i}G_{i}(z_{1}))$ , where $z_{1}$ is the number of particles present in Pool I at time t . Similarly, the hopping times between consecutive sites of lanes receiving inputs from Pool II are exponentially distributed with parameters $\eta_{1}^{i},\eta_{2}^{i},\ldots ,\eta_{p_{i}}^{i}$ the hopping time for the particle to hop to site 1 of lane i is calculated as $t+e(\lambda_{0}^{i}H_{i}(z_{2}))$ , where $z_{2}$ is the number of particles present in Pool II at time t . A simulation begins with an empty chain with all the particles distributed arbitrarily in the pools and continues for $10^{7}$ time steps. After removing the initial $10^{4}$ steps from the calculation, the steady-state density of each site is calculated as the number of time steps it was occupied divided by the overall simulation runtime. In the example below, we show that simulations match the model RFMNTP.

**Figure 7.**
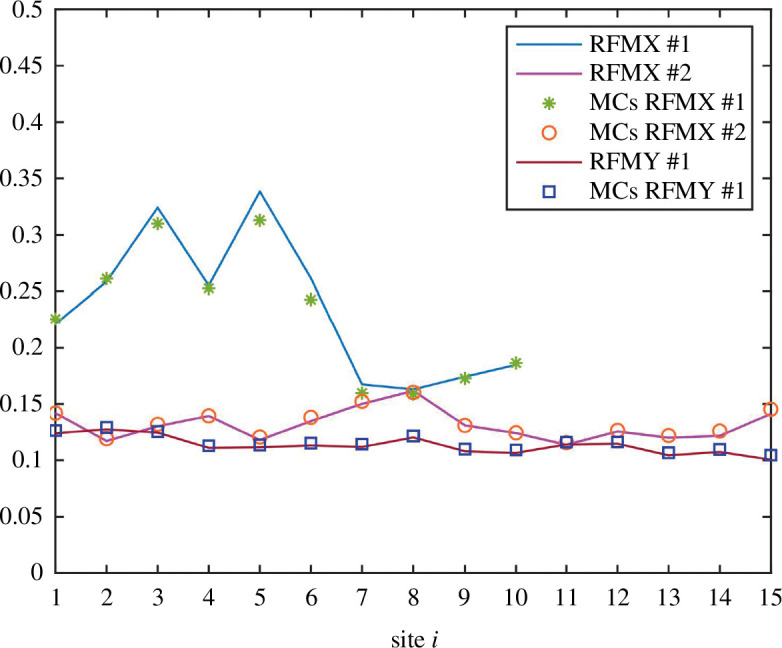
The steady-state density as a function of the site number for RFMXs and RFMYs in the RFMNTP in example 3.6. Solid lines and symbols denote numerically simulated RFMNTP and Monte Carlo simulations, respectively.

**Example 3.6** Consider an RFMNTP with m=2 RFMXs having dimensions $\ell_{1}=10$ , $\ell_{2}=15$ , n=1 RFMY having dimension $p_{1}=15$ , $\lambda_{0}^{i}=1$ , $\lambda_{j}^{1}=1+\theta_{j}$ , where $\theta_{j}$ is a random variable uniformly distributed in the interval (0,1) , $\lambda_{j}^{2}=2+\theta_{j}$ , $\eta_{0}^{1}=1$ , $\eta_{j}^{1}=5+\theta_{j}$ , $G_{i}(z_{1})=z_{1}$ , $H_{i}(z_{2})=z_{2}$ and first integral having value 7 . It can be observed in figure 7 that the steady-state density profile of the RFMNTP and the Monte Carlo simulations match well with each other.

## 4. Analysing a network with multiple pools

Consider a network consisting of M pools and N RFMs having interconnections via the pools. One can extend the analysis done in §3.1 to show that the network admits a continuum of steady-state points. The following example demonstrates the dynamic behaviour of the network with three pools.

**Figure 8.**
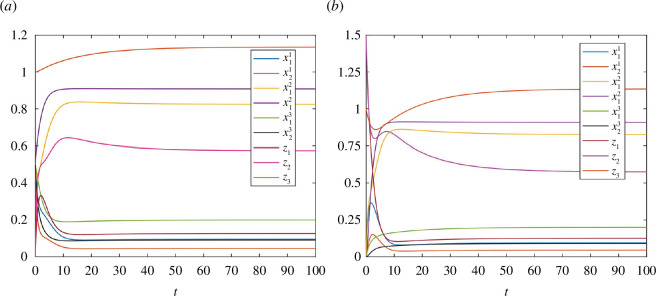
Trajectories of the network with three pools in example 4.1: (***a***) for initial condition $[0.5\;0.5\;0.5\;0.5\;0.5\;0.5\;0\;0\;1{]}^{\prime }$ and (***b***) for initial condition $[0\;0\;0\;0\;0\;0\;1.5\;1.5\;1{]}^{\prime }$ .

**Example 4.1.** Consider a network of three RFMs, each with dimension 2 , having three pools. Suppose RFM #1 receives its input from Pool #1 and supplies its output to Pool #2 , RFM #2 receives its input from Pool #2 and supplies its output to Pool #3 and RFM #3 receives its input from Pool #3 and supplies its output to Pool #1 . Assume that $\lambda_{0}^{1}=0.8$ , $\lambda_{1}^{1}=1$ , $\lambda_{2}^{1}=2$ , $\lambda_{0}^{2}=1$ , $\lambda_{1}^{2}=1.2$ , $\lambda_{2}^{2}=0.1$ , $\lambda_{0}^{3}=0.1$ , $\lambda_{1}^{3}=0.5$ , $\lambda_{2}^{3}=1$ , $G_{1}(z_{1})=\tanh(z_{1})$ , $G_{2}(z_{2})=\tanh(z_{2})$ and $G_{3}(z_{3})=z_{3}$ . There exists a unique equilibrium point e in $L_{4}$ and after calculation we have $e=[0.09514\;0.04541\;0.8249\;0.9082\;0.1998\;0.0908\;0.12613\;0.5745\;1.1350{]}^{\prime }$ . Figure 8*a,b* depicts trajectories of RFMNTP for initial conditions in the level set $L_{4}:\;[0.5\;0.5\;0.5\;0.5\;0.5\;0.5\;0\;0\;1{]}^{\prime }$ and $[0\;0\;0\;0\;0\;0\;1.5\;1.5\;1{]}^{\prime }$ , respectively. It can be observed that each of these trajectories converges to e .

Next, we understand the effect of modifying the transition rate of a site in an RFM on the entire network by analysing the steady-state densities. Equation (2.5) at steady-state e is given as


(3.21) $$\lambda_{0}^{k}G_{k}(e_{z_{j}})(1-e_{x_{1}}^{k})=\lambda_{1}^{k}e_{x_{1}}^{k}(1-e_{x_{2}}^{k})=\cdots =\lambda_{n_{k}}^{k}e_{x_{n_{k}}}^{k}.$$

Equation (2.6) at steady-state e is given as


(3.22) $$\sum_{k^{\prime }\in I^{\prime }}\lambda_{n_{k^{\prime }}}^{k^{\prime }}e_{x_{n_{k^{\prime }}}}^{k^{\prime }}=\sum_{k\in I}\lambda_{0}^{k}G_{k}(e_{z_{j}})(1-e_{x_{1}}^{k}).$$

Rewriting the above equation, we get


(3.23) $$\sum_{k^{\prime }\in I^{\prime }}\lambda_{n_{k^{\prime }}}^{k^{\prime }}e_{x_{n_{k^{\prime }}}}^{k^{\prime }}=\sum_{k\in I}\lambda_{n_{k}}^{k}e_{x_{n_{k}}}^{k}.$$

Without loss of generality, we assume that Pool #1 is feeding RFM #1 , and there is an increment in the rate $\lambda_{k}^{1}$ of RFM #1 . Let $\bar{e}$ represent the steady state of the modified network. Then by arguing similarly as in the proof of theorem 3.3, we get the information that ${\bar{e}}_{x_{k}}^{1}<e_{x_{k}}^{1}\;\text{and}\;e_{x_{j}}^{1}<{\bar{e}}_{x_{j}}^{1}\text{for all}\;j\in \{k+1,\ldots ,n_{1}\}$ . The steady-state densities of the RFMs and the pools associated directly with Pool #1 follow the cases mentioned in theorem 3.3 depending on the various parameter values. The change in the steady-state densities of the other pools depends on the total input it is receiving from the RFMs and can be analysed through equation (3.23). Also, the case when $e_{z_{1}}<{\bar{e}}_{z_{1}}$ and ${\bar{e}}_{z_{j}}<e_{z_{j}}$ for any j∈{1,2,…,M} is not possible as argued in theorem 3.3. This is a brief outline to gain an understanding of how the network with multiple pools behaves, as the exact scenario will be more clear when we know the interconnections between the RFMs via the pools.

In order to verify that the high correlation between the model and Monte Carlo simulations holds for a large set of parameters, we ran 250 tests, where in each test, a new set of rates are drawn randomly.

**Figure 9.**
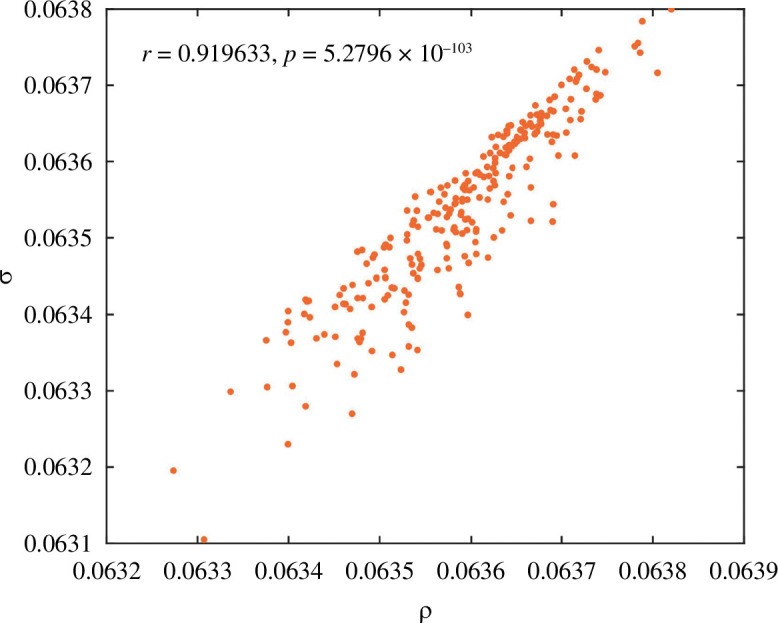
Steady-state mean densities (numerically simulated ρ and Monte Carlo simulated σ ) and the corresponding Pearson’s correlation coefficient r and p -value in example 4.2.

**Example 4.2.** Consider a network with M=3 pools and N=3 RFMs having dimensions $n_{1}=20$ , $n_{2}=30$ , $n_{3}=40$ , where RFM #1 /RFM #2 /RFM #3 receives its input from Pool #1 /Pool #2 /Pool #3 and supplies its output to Pool #2 /Pool #3 /Pool #1 . Assume that $\lambda_{0}^{i}=1$ , $\lambda_{j}^{1}=0.5+\theta_{j}$ , $\lambda_{j}^{2}=2+\theta_{j}$ , $\lambda_{j}^{3}=1+\theta_{j}$ , where $\theta_{j}$ is a random variable uniformly distributed in the interval (0,1) , $G_{i}(z_{j})=z_{j}$ and first integral having value 6 . Figure 9 depicts the correlations between the steady-state mean densities ( ρ ) of the RFMs and the steady-state mean densities ( σ ) calculated through Monte Carlo simulations. It can be seen that the correlation between the two is high ( r≃0.919633 ).

## 5. Discussion

Various transport phenomena involve the movement of particles along some tracks, for example, there is movement of RNA polymerases along DNA molecules, movement of ribosomes along mRNA molecules, motor proteins move along microtubules in order to transport cargo from one location to another, data packets move along buffers, there is the movement of vehicles along roads and many more. In all these systems, the particles are moving on a network of interconnected lanes in several transport systems. A common attribute in such phenomena is the presence of finite resources generating a closed network. The RFM was developed for analysing the excluded flow of particles along a one-dimensional isolated track, and it provides a useful and versatile modelling component that helps to understand the complex networks of the cellular as well as physical processes.

The network models consisting of a single pool are used to describe the behaviour of the system when the particles are distributed uniformly throughout the system. These single-pool models, however, do not take into account the distribution of particles in a local neighbourhood and, hence, are not able to model the movement of resources between different pools, for instance, the movement of cars (resources) between the two cities (pools). We introduced a new network model, RFMNTP, composed of several RFMs that focused on analysing how the network behaves when only the nearby resources impact the entry rates of its target. The RFMNTP is a closed network consisting of RFMs strategically connected to two pools such that Pool I (Pool II) feeds the input of some of the RFMs (remaining RFMs), and the output of them is fed into Pool II (Pool I). In other words, the first sites of some of the lanes and the last sites of the remaining lanes are connected to the same pool.

Understanding the stability of a system is a fundamental and foremost aspect of analysing systems in various fields of study. In this context, it is important to understand the stability of our network to predict its long-term behaviour. We prove that the RFMNTP is a cooperative irreducible dynamical system that admits a non-trivial first integral and, thus, enjoys several dynamical properties. In particular, the RFMNTP admits a continuum of steady-state points, and it entrains to periodic excitations in the parameters. Our theoretical analysis shows that an increase in the transition rate of a site in an RFM has a non-trivial effect on the output rates of the other RFMs. It can lead to any of the scenarios: the output rate of all the other RFMs increase or decrease; an increase in output rates of some of the RFMs and a decrease in output rates of the other RFMs. The specific outcome can be predicted by simulating the RFMNTP.

A noteworthy observation is that there could be a simultaneous increase in the output rates of some of the RFMs and a decrease in the output rates of the other RFMs. In the previous network model [25], we have seen that an increase in a transition rate in an RFM in the presence of a bottleneck rate leads to a decrease in the output rate of the other RFMs, whereas we can see in example 3.4 that this may not hold owing to aspect of local sharing of particles incorporated through two pools. Next, we have illustrated how to gain an understanding of how the changes in an RFM affect other RFMs and the overall behaviour of the network with multiple pools.

The model described here can be generalized to capture more complicated features. For example, the output of the shorter lanes can be fed back into the same pool. This phenomenon may be studied by adding its output rate to the same pool. The RFM also provides an analytical framework for modelling and analysing linear communication networks [64]. In this context, the moving particles are data packets, the chain of sites is a one-dimensional chain of ordered buffers, and the decreasing entry rate to a fuller buffer represents a kind of decentralized backpressure flow control. Another research direction is to analyse networks, comprising multiple flows that share common nodes, using a set of interconnected RFMs, constraining the link capacities in the communication networks. An applicability of our model can be to analyse a network topology where a common source node is linked to several chains of ordered buffers. The output of these chains at the destination node can be a source node for other sets of chains of ordered buffers and so on. One may also generalize RFMNTP by considering nearest-neighbour interactions in the network as seen in molecular motor traffic. Another interesting direction is to try and validate our predictions about the local behaviour of the cellular environment experimentally.

## Data Availability

This article has no additional data.

## Appendix A. Proofs

*Proof of Proposition 3.2*: For simplicity, we consider RFMNTP with one RFMX and one RFMY. Let $x_{j}^{1}:=x_{j}$ and $y_{j}^{1}:=y_{j}$ . Also, let $x_{0}:=z_{1}$ , $x_{\ell +1}:=z_{2}$ , $x_{\ell +j+1}:=y_{j}$ for j=1,2,…,p and $x_{-1}:=x_{\ell +p+1}$ . We now show that the system with state-variables $x_{0},\ldots ,x_{\ell +p+1}$ satisfies the cyclic boundary-repelling (CBR) property given in [25] (i.e. for any δ>0 and any sufficiently small △>0 , ∃ P=P(δ,△)>0 such that for each k=1,…,ℓ+p+1 and each t≥0 the condition $x_{k}(t)\leq △\text{ and }x_{k-1}(t)\geq \delta$ implies that ${\dot{x}}_{k}\geq P$ ). For k=0 , we have ${\dot{x}}_{0}=\eta_{p}x_{\ell +p+1}-\lambda_{0}G(x_{0})(1-x_{1})\geq \eta_{p}\delta -\lambda_{0}G(△)(1-x_{1})$ , and we have G(0)=0 , G is a continuous function and thus ${\dot{x}}_{0}\geq \eta_{p}\delta /2$ for all △>0 sufficiently small. Now for k=1 , we have ${\dot{x}}_{1}=\lambda_{0}G(z_{1})(1-x_{1})-\lambda_{1}x_{1}(1-x_{2})\geq \lambda_{0}G(\delta )(1-△)-\lambda_{1}△(1-x_{2})$ , so ${\dot{x}}_{1}\geq \lambda_{0}G(\delta )/2$ for all △>0 sufficiently small. For k∈{2,…,ℓ} , we have ${\dot{x}}_{k}=\lambda_{k-1}x_{k-1}(1-x_{k})-\lambda_{k}x_{k}(1-x_{k+1})\geq \lambda_{k}\delta (1-△)-\lambda_{k}△(1-x_{k+1})$ , and therefore, ${\dot{x}}_{k}\geq \lambda_{k}\delta /2$ for all △>0 sufficiently small. For k=ℓ+1 , we have ${\dot{x}}_{\ell +1}=\lambda_{\ell }x_{\ell }-\eta_{0}H(x_{\ell +1})(1-x_{\ell +2})\geq \lambda_{\ell }\delta -\eta_{0}H(△)(1-x_{\ell +2})$ , and we have H(0)=0 , H is a continuous function and thus ${\dot{x}}_{\ell +1}\geq \lambda_{\ell }\delta /2$ for all △>0 sufficiently small. Now, for k=ℓ+2 , we have ${\dot{x}}_{\ell +2}=\eta_{0}H(z_{2})(1-x_{\ell +2})-\eta_{1}x_{\ell +2}(1-x_{\ell +3})\geq \eta_{0}H(\delta )(1-△)-\eta_{1}△(1-x_{\ell +3})$ , so ${\dot{x}}_{\ell +2}\geq \eta_{0}H(\delta )/2$ for all △>0 sufficiently small. For k∈{ℓ+3,…,ℓ+p+1} , we have ${\dot{x}}_{k}=\eta_{k-1}x_{k-1}(1-x_{k})-\eta_{k}x_{k}(1-x_{k+1})\geq \eta_{k}\delta (1-△)-\eta_{k}△(1-x_{k+1})$ , and therefore, ${\dot{x}}_{k}\geq \eta_{k}\delta /2$ for all △>0 sufficiently small. Thus, the RFMNTP satisfies CBR.

Also, observe that if $x_{k}(t)>0$ and t>0 then $x_{k}(T)>0$ for all T≥t . It now follows from the result on repelling boundaries and persistence in ([25], lemma 1) that for any τ>0 , there exists $\epsilon_{1}(\tau )>0$ , with $\epsilon_{1}(\tau )\to 0$ as τ→0 , such that for any non-zero initial condition and any t≥τ the solution of the RFMNTP satisfies

(A 1)
$$\epsilon_{1}\leq x_{i}(t),\;\text{ for all }i\in \{0,\ldots ,\ell +p+1\}.$$

For the other part of the equality, define $u_{i}(t):=1-x_{\ell +1-i}(t)$ for i=1,2,…,ℓ and $v_{i}(t):=1-y_{p+1-i}(t)$ for i=1,2,…,p . From equation (A 1), we have $z_{1}\geq \epsilon_{1}$ and $z_{2}\geq \epsilon_{1}$ for all t≥τ . Note that the system with state variables $u_{1},u_{2},\ldots ,u_{\ell }$ is an RFM having time-varying exit rate $\lambda_{0}G(z_{1})$ and the system with state variables $v_{1},v_{2},\ldots ,v_{p}$ is an RFM having time-varying exit rate $\eta_{0}H(z_{2})$ . Therefore, $\exists \;\epsilon_{2}(\tau )>0$ and $\epsilon_{3}(\tau )>0$ such that any t≥τ , we have $\epsilon_{2}\leq u_{i}(t),\text{ for all }i\in \{1,\ldots ,\ell \}$ and $\epsilon_{3}\leq v_{i}(t),\text{ for all }i\in \{1,\ldots ,p\}$ . Combining this with the definition of $u_{i}$ and $v_{i}$ completes the proof.∎

*Proof of Theorem 3.1*: We have that the RFMNTP is a cooperative irreducible system on Int(B) with a non-trivial first integral. Combining this with proposition 3.2 and the results in ([54], theorems 10 and 11) completes the proof of this theorem.∎

*Proof of Theorem 3.2*: From proposition 3.2, it follows that $\phi_{r}\in \text{Int}(B)$ , and the results in [53] (see theorem 3.1) prove this theorem.∎

*Proof of Theorem 3.3*: At steady-state, we have

(A 2)
$$\sum_{i=1}^{m}\sum_{j=1}^{\ell_{i}}e_{x_{j}}^{i}+\sum_{i=1}^{n}\sum_{j=1}^{p_{i}}e_{y_{j}}^{i}+e_{z_{1}}+e_{z_{2}}=r,$$

and this also holds for the modified network since the initial condition is the same, that is,

(A 3)
$$\sum_{i=1}^{m}\sum_{j=1}^{\ell_{i}}{\bar{e}}_{x_{j}}^{i}+\sum_{i=1}^{n}\sum_{j=1}^{p_{i}}{\bar{e}}_{y_{j}}^{i}+{\bar{e}}_{z_{1}}+{\bar{e}}_{z_{2}}=r.$$

Pick $k\in \{1,2,...\ell_{1}-1\}$ . Let us assume that

(A 4)
$${\bar{e}}_{x_{\ell_{1}}}^{1}\leq e_{x_{\ell_{1}}}^{1}.$$

Then equation (3.7) implies that

(A 5)
$$\lambda_{\ell_{1}-1}^{1}{\bar{e}}_{x_{\ell_{1}-1}}^{1}(1-{\bar{e}}_{x_{\ell_{1}}}^{1})\leq \lambda_{\ell_{1}-1}^{1}e_{x_{\ell_{1}-1}}^{1}(1-e_{x_{\ell_{1}}}^{1}).$$

From equation (A 4), the above equation implies that ${\bar{e}}_{x_{\ell_{1}-1}}^{1}\leq e_{x_{\ell_{1}-1}}^{1}.$ Continuing this way, we have

(A 6)
$${\bar{e}}_{x_{j}}^{1}\leq e_{x_{j}}^{1}\;\;\text{for all}\;j=k+1,k+2,\ldots ,\ell_{j}.$$

Now, from equation (3.7), consider

(A 7)
$${\bar{\lambda }}_{k}^{1}{\bar{e}}_{x_{k}}^{1}(1-{\bar{e}}_{x_{k+1}}^{1})\leq \lambda_{k}^{1}e_{x_{k}}^{1}(1-e_{x_{k+1}}^{1}).$$

We have $\lambda_{k}^{1}<{\bar{\lambda }}_{k}^{1}$ , thereby equation (A 7) implies that

(A 8)
$${\bar{\lambda }}_{k}^{1}{\bar{e}}_{x_{k}}^{1}(1-{\bar{e}}_{x_{k+1}}^{1})<{\bar{\lambda }}_{k}^{1}e_{x_{k}}^{1}(1-e_{x_{k+1}}^{1}),$$

which implies ${\bar{e}}_{x_{k}}^{1}<e_{x_{k}}^{1}$ . From equation (3.7), we have

(A 9)
$$\lambda_{k-1}^{1}{\bar{e}}_{x_{k-1}}^{1}(1-{\bar{e}}_{x_{k}}^{1})<\lambda_{k-1}^{1}e_{x_{k-1}}^{1}(1-e_{x_{k}}^{1}).$$

Now, since ${\bar{e}}_{x_{k}}^{1}<e_{x_{k}}^{1}$ , we must have ${\bar{e}}_{x_{k-1}}^{1}<e_{x_{k-1}}^{1}$ . Continuing in this way, we get

(A 10)
$${\bar{e}}_{x_{j}}^{1}<e_{x_{j}}^{1}\;\;\text{for all}\;j=1,2,\ldots ,k-2.$$

This also implies that ${\bar{e}}_{z_{1}}<e_{z_{1}}$ . Since RFMX is a monotone control system, and therefore we have

(A 11)
$${\bar{e}}_{x_{\ell_{j}}}^{i}<e_{x_{\ell_{j}}}^{i}\;\;\text{for all}\;i=2,3,\ldots ,m\;\;\text{and}\;j=1,2,\ldots ,\ell_{i}.$$

Note that all the RFMYs are interconnected through the pool variable $z_{2}$ , and hence, all are affected in the same manner. From equations (3.10), (A 4) and (A 11), we have

(A 12)
$${\bar{e}}_{y_{\ell_{j}}}^{i}<e_{y_{\ell_{j}}}^{i}\;\;\text{for all}\;i=1,2,\ldots ,n\;\;\text{and}\;j=1,2,\ldots ,p_{i},$$

and this implies ${\bar{e}}_{z_{2}}<e_{z_{2}}$ , which yields the contradiction to the equation (A 3). Hence, $e_{x_{\ell_{1}}}^{1}<{\bar{e}}_{x_{\ell_{1}}}^{1}$ . Now if $e_{x_{k}}^{1}\leq {\bar{e}}_{x_{k}}^{1}$ , this implies that $e_{z_{1}}<{\bar{e}}_{z_{1}}$ which further implies $e_{y_{\ell_{j}}}^{i}<{\bar{e}}_{y_{\ell_{j}}}^{i}\;\text{for all}\;i=1,2,\ldots ,n\;\text{and}\;j=1,2,\ldots ,p_{i}$ and $e_{z_{2}}<{\bar{e}}_{z_{2}}$ . This is again a contradiction to equation (A 3). Also, note that the case $e_{z_{1}}$ is increasing and $e_{z_{2}}$ is decreasing is not possible as this will lead to the contradiction to equation (3.10). Now, consider k=0 . Let us assume that

(A 13)
$${\bar{e}}_{x_{\ell_{1}}}^{1}\leq e_{x_{\ell_{1}}}^{1}.$$

Continuing as above, we have

(A 14)
$${\bar{e}}_{x_{j}}^{1}\leq e_{x_{j}}^{1}\;\;\text{for all}\;j=1,2,\ldots ,\ell_{1}-1.$$

Now, from equation (3.7), consider

(A 15)
$$\lambda_{0}^{1}G_{1}({\bar{e}}_{z_{1}})(1-{\bar{e}}_{x_{1}}^{1})\leq \lambda_{0}^{1}G_{1}(e_{z_{1}})(1-e_{x_{1}}^{1}).$$

We have $\lambda_{0}^{1}<{\bar{\lambda }}_{0}^{1}$ and thereby equations (A 14) and (A 15) imply ${\bar{e}}_{z_{1}}<e_{z_{1}}$ . Again using the above arguments, we get the contradiction to equation (A 13).

Now, consider $k=\ell_{1}$ . Then, we have to show that $e_{x_{\ell_{1}}}^{-1}<e_{x_{\ell_{1}}}^{1}$ . Seeking a contradiction, assume

(A 16)
$$e_{x_{\ell_{1}}}^{1}\leq {\bar{e}}_{x_{\ell_{1}}}^{1}.$$

Then, equation (3.7) with the fact that $\lambda_{\ell_{1}}^{1}<{\bar{\lambda }}_{\ell_{1}}^{1}$ implies that

(A 17)
$$\lambda_{\ell_{1}-1}^{1}e_{x_{\ell_{1}-1}}^{1}(1-e_{x_{\ell_{1}}}^{1})<\lambda_{\ell_{1}-1}^{1}{\bar{e}}_{x_{\ell_{1}-1}}^{1}(1-{\bar{e}}_{x_{\ell_{1}}}^{1}).$$

From equation (A 16), the above equation implies that $e_{x_{\ell_{1}-1}}^{1}<{\bar{e}}_{x_{\ell_{1}-1}}^{1}.$ Continuing this way, we have

(A 18)
$$e_{x_{j}}^{1}<{\bar{e}}_{x_{j}}^{1}\;\;\text{for all}\;j=1,2,\ldots ,\ell_{1}-2.$$

This also implies that $e_{z_{1}}<{\bar{e}}_{z_{1}}$ . Since RFMX is a monotone control system, and therefore, we have

(A 19)
$$e_{x_{\ell_{j}}}^{i}<{\bar{e}}_{x_{\ell_{j}}}^{i}\;\;\text{for all}\;i=2,3,\ldots ,m\;\;\text{and}\;j=1,2,\ldots ,\ell_{i}.$$

Note that all the RFMYs are interconnected through the pool variable $z_{2}$ , and hence all are affected in the same manner. From equations (3.10), (A 16) and (A 19) and the fact that $\lambda_{\ell_{1}}^{1}<{\bar{\lambda }}_{\ell_{1}}^{1}$ , we have

(A 20)
$$e_{y_{\ell_{j}}}^{i}<{\bar{e}}_{y_{\ell_{j}}}^{i}\;\;\text{for all}\;i=1,2,\ldots ,n\;\;\text{and}\;j=1,2,\ldots ,p_{i},$$

and this implies $e_{z_{2}}<{\bar{e}}_{z_{2}}$ , which yields a contradiction to equation (A 3). Hence, ${\bar{e}}_{x_{\ell_{1}}}^{1}<e_{x_{\ell_{1}}}^{1}$ . This completes the proof of this theorem.∎

*Proof of Proposition 3.3*: The network considered in part (a) has identical RFMXs, and hence, the steady-state density profile of the RFMXs are the same, and similarly, this holds for RFMYs. Without loss of generality, consider the steady-state equations for RFMX #1 in (a)

(A 21)
$$\lambda_{0}G(e_{z_{1}})(1-e_{x_{1}}^{1})=\lambda_{1}e_{x_{1}}^{1}(1-e_{x_{2}}^{1})=\cdots =\lambda_{\ell }e_{x_{\ell }}^{1},$$

and the steady-state equation for RFMY #1 in (a) is as follows:

(A 22)
$$\eta_{0}H(e_{z_{2}})(1-e_{y_{1}}^{1})=\eta_{1}e_{y_{1}}^{1}(1-e_{y_{2}}^{1})=\cdots =\eta_{p}e_{y_{p}}^{1}.$$

Similarly, the steady-state equation for RFMX in (b)

(A 23)
$$m\lambda_{0}G({\tilde{e}}_{z_{1}})(1-{\tilde{e}}_{x_{1}})=\lambda_{1}{\tilde{e}}_{x_{1}}(1-{\tilde{e}}_{x_{2}})=\cdots =\lambda_{\ell }{\tilde{e}}_{x_{\ell }},$$

and the steady-state equation for the RFMY in (b) is as follows:

(A 24)
$$m\eta_{0}H({\tilde{e}}_{z_{2}})(1-{\tilde{e}}_{y_{1}})=\eta_{1}{\tilde{e}}_{y_{1}}(1-{\tilde{e}}_{y_{2}})=\cdots =\eta_{p}{\tilde{e}}_{y_{p}}.$$

Now, consider equations (A 21) and (A 23); suppose we have

(A 25)
$$\lambda_{\ell }e_{x_{\ell }}^{1}=\lambda_{\ell }{\tilde{e}}_{x_{\ell }}$$

(A 26)
$$\;⟹\;e_{x_{i}}^{1}={\tilde{e}}_{x_{i}}\;\text{for all}\;i$$

and also

(A 27)
$$\lambda_{0}G(e_{z_{1}})(1-e_{x_{1}}^{1})=m\lambda_{0}G({\tilde{e}}_{z_{1}})(1-{\tilde{e}}_{x_{1}})$$

(A 28)
$$\;⟹\;e_{z_{1}}=m{\tilde{e}}_{z_{1}}.$$

Similarly, we get

(A 29)
$$e_{y_{i}}^{1}={\tilde{e}}_{y_{i}}\;\;\text{for all}\;\;i\;\text{and}\;e_{z_{2}}=m{\tilde{e}}_{z_{2}}.$$

Now, steady-state equation (3.5) for the network (a) is

(A 30)
$$m(e_{x_{1}}^{1}+e_{x_{2}}^{1}+\cdots +e_{x_{\ell }}^{1})+m(e_{y_{1}}^{1}+e_{y_{2}}^{1}+\cdots +e_{y_{p}}^{1})+e_{z_{1}}+e_{z_{2}}=r.$$

By equations (A 26), (A 28) and (A 29), we get

(A 31)
$${\tilde{e}}_{x_{1}}+{\tilde{e}}_{x_{2}}+\cdots +{\tilde{e}}_{x_{\ell }}+{\tilde{e}}_{y_{1}}+{\tilde{e}}_{y_{2}}+\cdots +{\tilde{e}}_{y_{p}}+{\tilde{e}}_{z_{1}}+{\tilde{e}}_{z_{2}}=r/m.$$

and hence, we get the steady-state equation for the network (b), and this completes the proof of the proposition.∎
